# Supplementary figures and images for: Regulatory logic of endogenous RNAi in silencing de novo genomic conflicts
Source: PLoS Genet. 2023 Jun 21;19(6):e1010787. doi: 10.1371/journal.pgen.1010787 (PMC10317233; doi:10.1371/journal.pgen.1010787)

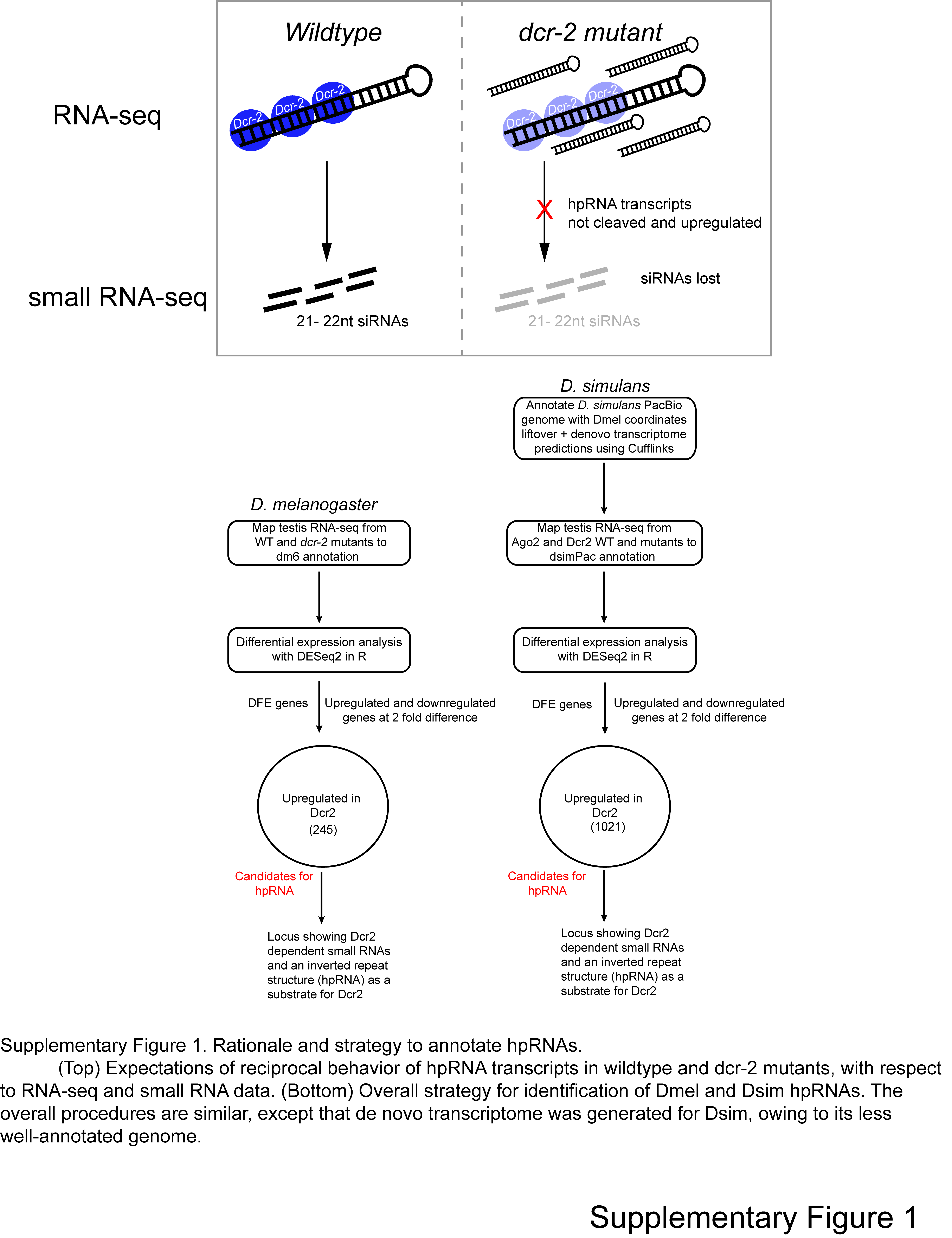

Supplement: S1 Fig — (Top) Expectations of reciprocal behavior of hpRNA transcripts in wildtype and dcr-2 mutants, with respect to RNA-seq and small RNA data. (Bottom) Overall strategy for identification of D. melanogaster and D. simulans hpRNAs. The overall procedures are similar, except that de novo transcriptome was generated for D. simulans, owing to its less well-annotated genome. (TIF) [file pgen.1010787.s001.tif]

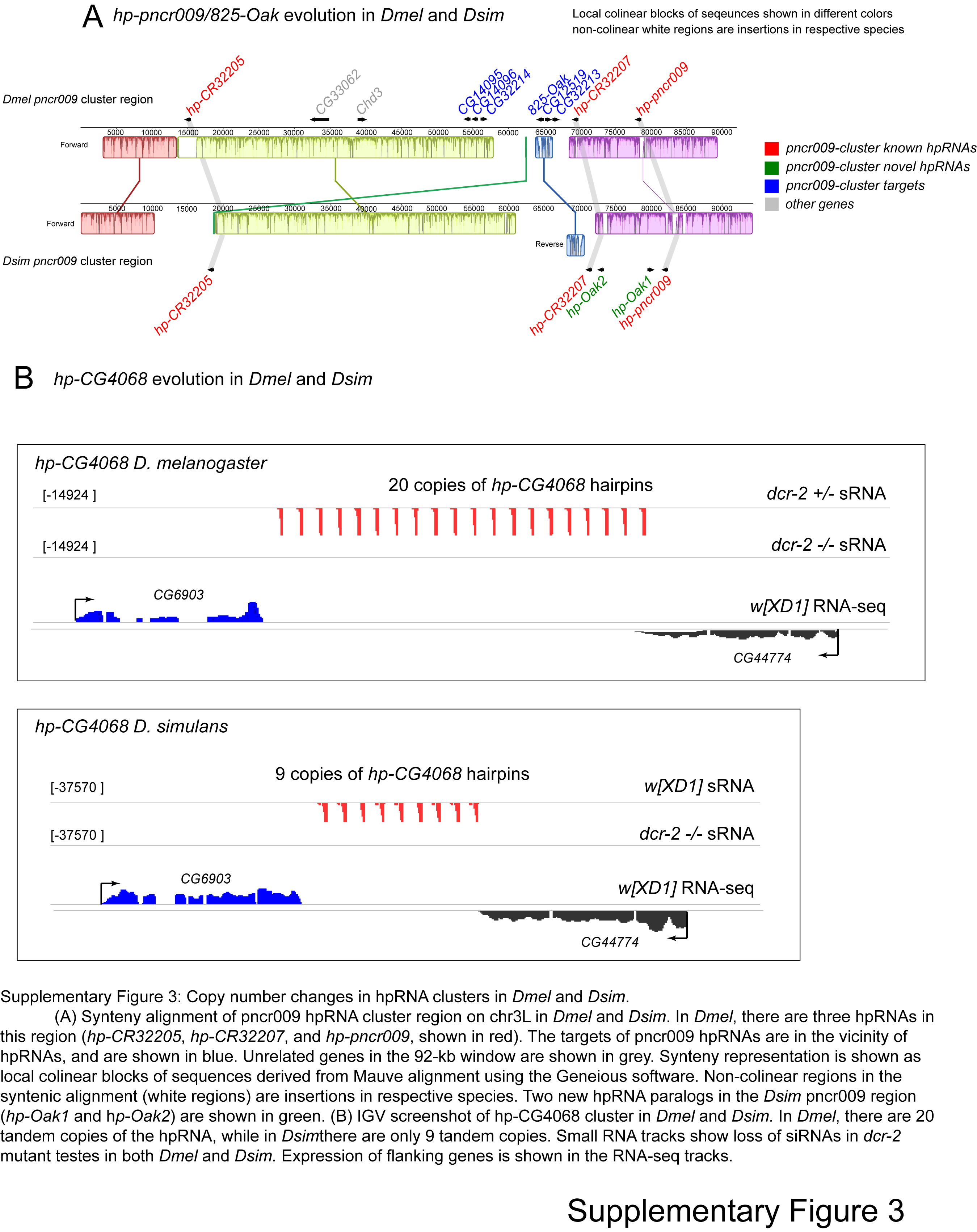

Supplement: S3 Fig — (A) Synteny alignment of pncr009 hpRNA cluster region on chr3L in D. melanogaster and D. simulans. In D. melanogaster, there are three hpRNAs in this region (hp-CR32205, hp-CR32207, and hp-pncr009, shown in red). The targets of pncr009 hpRNAs are in the vicinity of hpRNAs, and are shown in blue. Unrelated genes in the 92-kb window are shown in grey. Synteny representation is shown as local colinear blocks of sequences derived from Mauve alignment using the Geneious software. Non-colinear regions in the syntenic alignment (white regions) are insertions in respective species. Two novel hpRNA paralogs in the D. simulans pncr009 region (hp-Oak1 and hp-Oak2) are shown in green. (B) IGV screenshot of hp-CG4068 cluster in D. melanogaster and D. simulans. In D. melanogaster, there are 20 tandem copies of the hpRNA, while in D. simulans there are only 9 tandem copies. Small RNA tracks show loss of siRNAs in dcr-2 mutant testes in both D. melanogaster and D. simulans. Expression of flanking genes is shown in the RNA-seq tracks. (TIF) [file pgen.1010787.s003.tif]

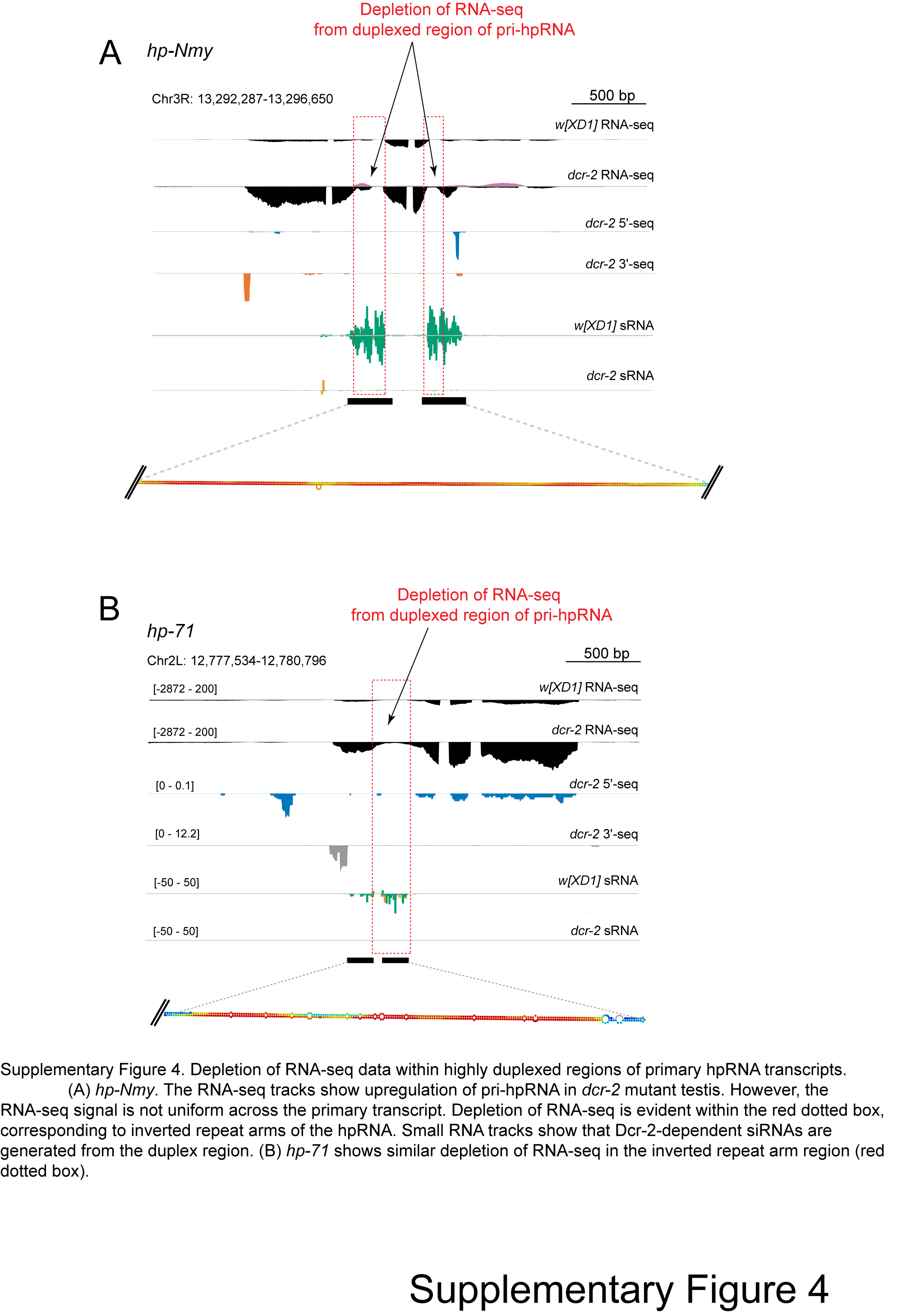

Supplement: S4 Fig — (A) hp-Nmy. The RNA-seq tracks show upregulation of pri-hpRNA in dcr-2 mutant testis. However, the RNA-seq signal is not uniform across the primary transcript. Depletion of RNA-seq is evident within the red dotted box, corresponding to inverted repeat arms of the hpRNA. Small RNA tracks show that Dcr-2-dependent siRNAs are generated from the duplex region. (B) hp-71 shows similar depletion of RNA-seq in the inverted repeat arm region (red dotted box). (TIF) [file pgen.1010787.s004.tif]

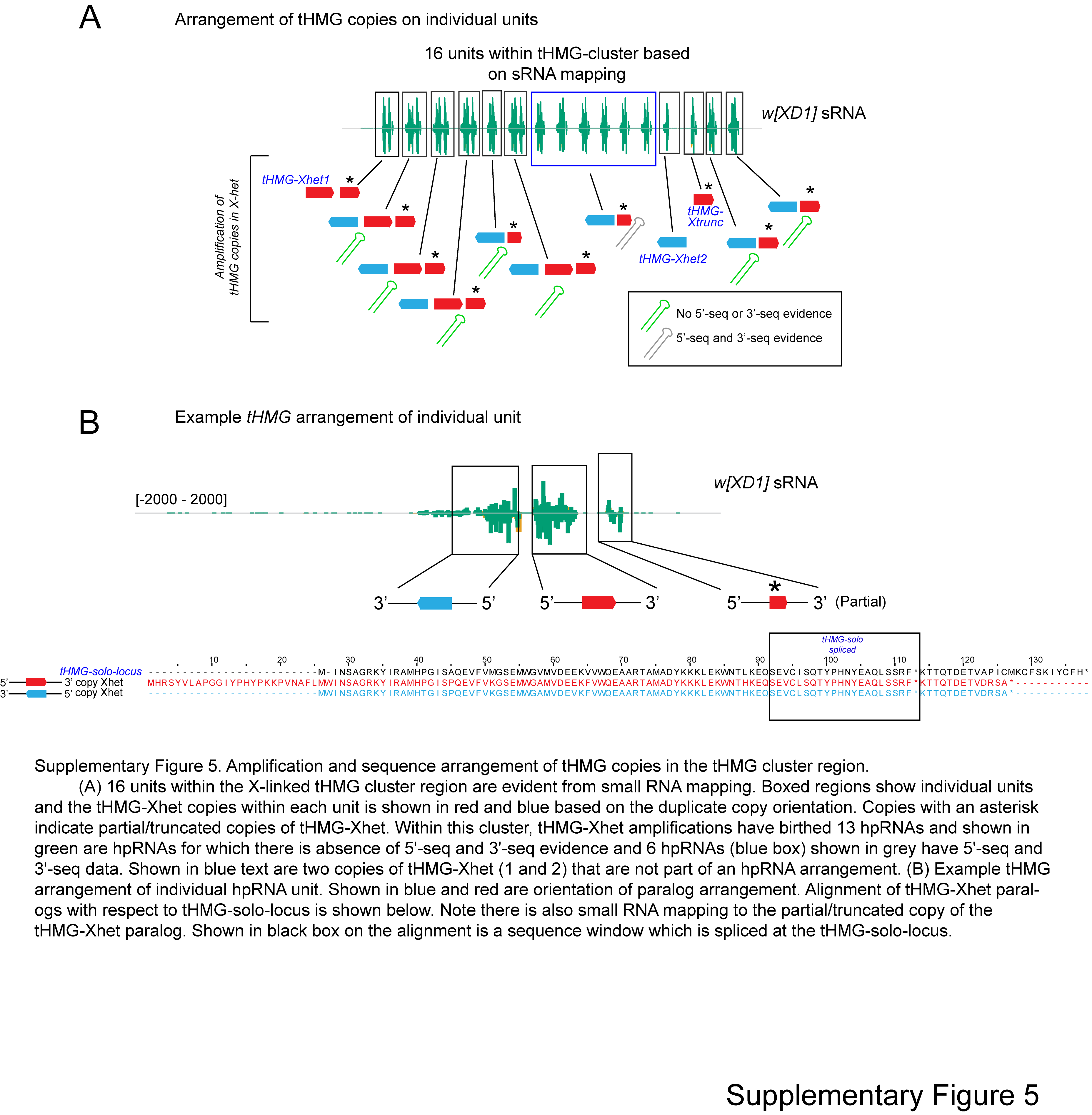

Supplement: S5 Fig — (A) 16 units within the X-linked tHMG cluster region are evident from small RNA mapping. Boxed regions show individual units and the tHMG-Xhet copies within each unit is shown in red and blue based on the duplicate copy orientation. Copies with an asterisk indicate partial/truncated copies of tHMG-Xhet. Within this cluster, tHMG-Xhet amplifications have birthed 13 hpRNAs and shown in green are hpRNAs for which there is absence of 5’-seq and 3’-seq evidence and 6 hpRNAs (blue box) shown in grey have 5’-seq and 3’-seq data. Shown in blue text are two copies of tHMG-Xhet (1 and 2) that are not part of an hpRNA arrangement. (B) Example tHMG arrangement of individual hpRNA unit. Shown in blue and red are orientation of paralog arrangement. Alignment of tHMG-Xhet paralogs with respect to tHMG-solo-locus is shown below. Note there is also small RNA mapping to the partial/truncated copy of the tHMG-Xhet paralog. Shown in black box on the alignment is a sequence window which is spliced at the tHMG-solo-locus. (TIF) [file pgen.1010787.s005.tif]

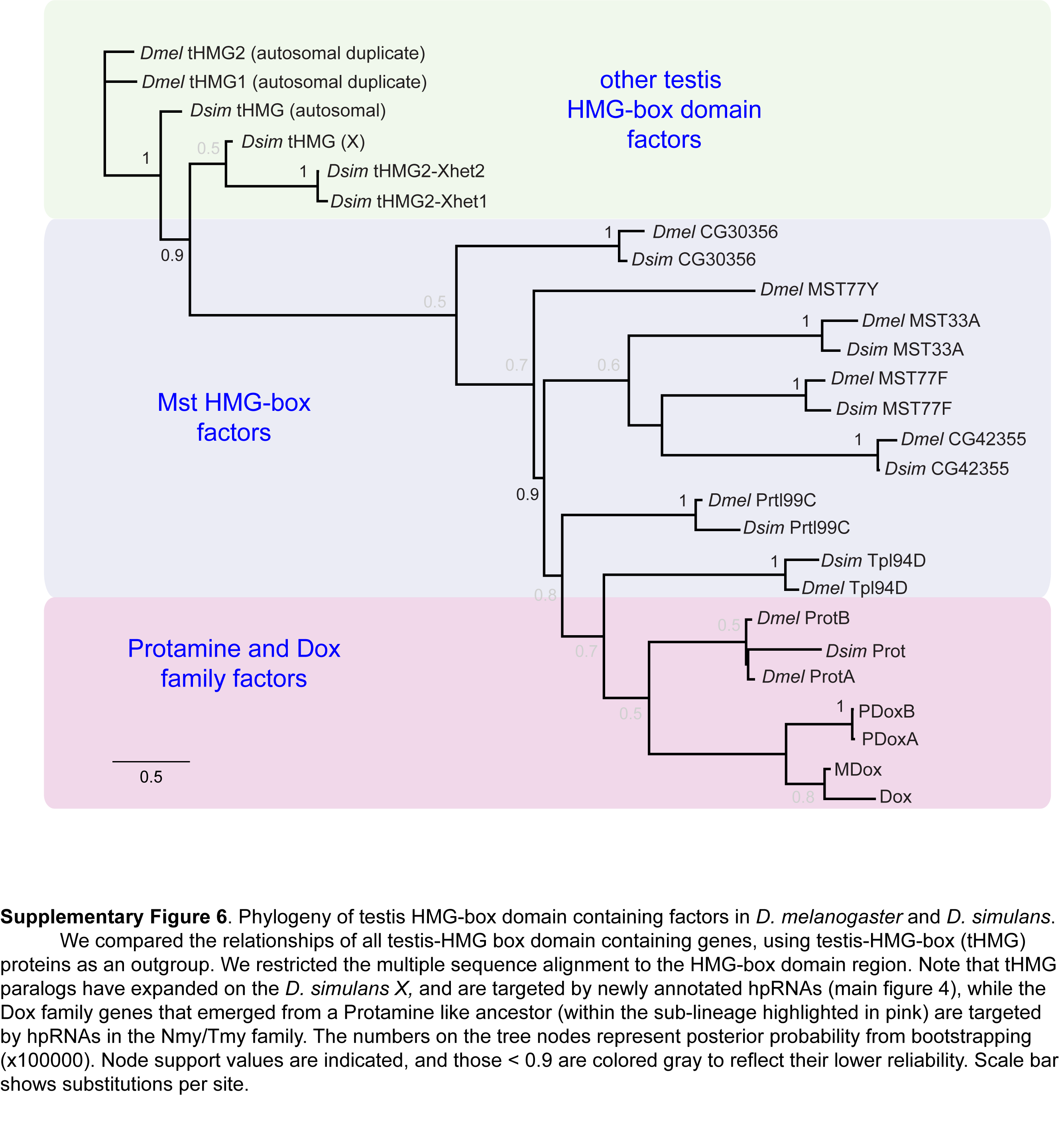

Supplement: S6 Fig — HMG-box domain containing loci with testis-specific expression was identified in [73]. We compared the relationships of all testis-HMG-box domain containing genes, using testis HMG-box domain (tHMG) proteins as an outgroup. tHMG paralogs in D. simulans are targeted by hpRNAs (main Fig 4), while the Dox family genes that emerged from Protamine like ancestor (ODox, red rectangle) are targeted by hp-Nmy and hp-Tmy. In contemporary D. simulans, ODox and its duplicate are distinct hpRNAs (main Fig 4). (TIF) [file pgen.1010787.s006.tif]

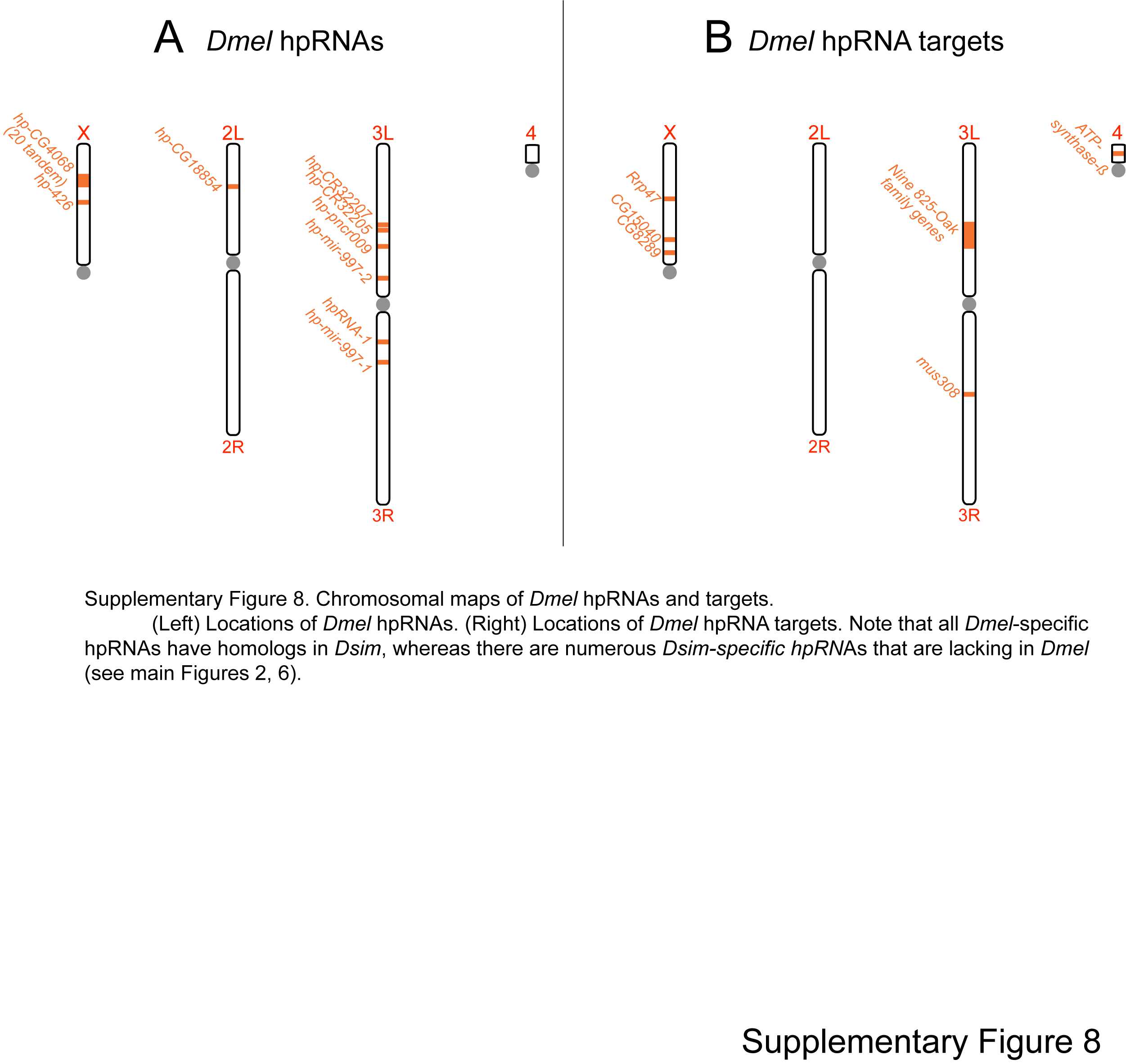

Supplement: S8 Fig — (Left) Locations of D. melanogaster hpRNAs. (Right) Locations of D. melanogaster hpRNA targets. Note that all D. melanogaster-specific hpRNAs have homologs in D. simulans, whereas there are numerous D. simulans-specific hpRNAs that are lacking in D. melanogaster (see main Figs 2 and 6). (TIF) [file pgen.1010787.s008.tif]
