# Supplementary material for: Regulatory logic of endogenous RNAi in silencing de novo genomic conflicts
Source: PLoS Genet. 2023 Jun 21;19(6):e1010787. doi: 10.1371/journal.pgen.1010787 (PMC10317233; doi:10.1371/journal.pgen.1010787)
Supplement: Attachment — Submitted filename: 2023_0426_Dsim_hpRNA_expansion_rev_responses.pdf [file pgen.1010787.s014.pdf]

Dear Dr Lai,

Thank you very much for submitting your Research Article entitled 'Regulatory logic of endogenous RNAi in silencing de novo genomic conflicts' to PLOS Genetics.
The manuscript was fully evaluated at the editorial level and by independent peer reviewers. The reviewers appreciated the attention to an important topic but identified some concerns that we ask you address in a revised manuscript.

We therefore ask you to modify the manuscript according to the review recommendations. Your revisions should address the specific points made by each reviewer.

In addition we ask that you:
1) Provide a detailed list of your responses to the review comments and a description of the changes you have made in the manuscript.
2) Upload a Striking Image with a corresponding caption to accompany your manuscript if one is available (either a new image or an existing one from within your manuscript). If this image is judged to be suitable, it may be featured on our website. Images should ideally be high resolution, eye-catching, single panel square images. For examples, please browse our archive. If your image is from someone other than yourself, please ensure that the artist has read and agreed to the terms and conditions of the Creative Commons Attribution License. Note: we cannot publish copyrighted images.
We hope to receive your revised manuscript within the next 30 days. If you anticipate any delay in its return, we would ask you to let us know the expected resubmission date by email to plosgenetics@plos.org.

If present, accompanying reviewer attachments should be included with this email; please notify the journal office if any appear to be missing. They will also be available for download from the link below. You can use this link to log into the system when you are ready to submit a revised version, having first consulted our Submission Checklist.

While revising your submission, please upload your figure files to the Preflight Analysis and Conversion Engine (PACE) digital diagnostic tool. PACE helps ensure that figures meet PLOS requirements. To use PACE, you must first register as a user. Then, login and navigate to the UPLOAD tab, where you will find detailed instructions on how to use the tool. If you encounter any issues or have any questions when using PACE, please email us at figures@plos.org.

Please be aware that our data availability policy requires that all numerical data underlying graphs or summary statistics are included with the submission, and you will need to provide this upon resubmission if not already present. In addition, we do not permit the inclusion of phrases such as "data not shown" or "unpublished results" in manuscripts. All points should be backed up by data provided with the submission.
To enhance the reproducibility of your results, we recommend that you deposit your laboratory protocols in protocols.io, where a protocol can be assigned its own identifier (DOI) such that it can be cited independently in the future. Additionally, PLOS ONE offers an option to publish peer-reviewed clinical study protocols. Read more information on sharing protocols at https://plos.org/protocols?utm_medium=editorial-email&utm_source=authorletters&utm_campaign=protocols
Please review your reference list to ensure that it is complete and correct. If you have cited papers that have been retracted, please include the rationale for doing so in the manuscript text, or remove these references and replace them with relevant current references. Any changes to the reference list should be mentioned in the rebuttal letter that accompanies your revised manuscript. If you need to cite a retracted article, indicate the article's retracted status in the References list and also include a citation and full reference for the retraction notice.
PLOS has incorporated Similarity Check, powered by iThenticate, into its journal-wide submission system in order to screen submitted content for originality before publication. Each PLOS journal undertakes screening on a proportion of submitted articles. You will be contacted if needed following the screening process.

To resubmit, you will need to go to the link below and 'Revise Submission' in the 'Submissions Needing Revision' folder.

Please let us know if you have any questions while making these revisions.

Yours sincerely,

A. Aziz Aboobaker
Academic Editor
PLOS Genetics

Bret Payseur
Section Editor
PLOS Genetics

Reviewer's Responses to Questions
Comments to the Authors:
Please note here if the review is uploaded as an attachment.
Reviewer #1: Overall- This is an interesting manuscript discussing a specific type of small RNA, known as hpRNA, which is dependent on the Drosophila dicer paralogue DCR-2. The authors characterise this type of RNA in Drosophila simulans dcr-2 mutants, demonstrating clear deregulation. They then characterise targets of hpRNAs and their rough "age" and make some speculations about how this may link to genomic conflicts, although these are not really backed up by any data. I think it shows some new insights into what sort of genes hpRNAs target and provides insights into the diversity of different types of small RNAs across evolution as hpRNAs seem to be restricted to arthropods. I have a few small comments about the analyses performed which the authors might want to consider (major points) and a few text suggestions (minor comments).

We thank the reviewer for the positive comments on the manuscript. Selfish genetic elements are ubiquitous across the metazoan tree of life, and mechanisms to counteract intragenomic conflicts /selfish genes, particularly in the germline, have recurrently come about in many ways. One such evolutionary innovation is the hpRNA/RNAi-mediated defense. Our foray into the comparative genomic analyses of RNAi effector *dcr2* revealed the hpRNA/RNAi pathway is crucial in mitigating sex chromosome conflict, mediated by the *Dox* family genes that emerged within the *simulans* clade of *Drosophila* species.

We agree with the reviewer that besides the *Dox* family genes, evidence for whether or not other novel targets of RNAi may be embroiled in an intragenomic conflict remain to be experimentally tested ("*they...make some speculations about how this may link to genomic conflicts, although these are not really backed up by any data*"). However, the key point of this study is that there is currently no other extant forward strategy that can identify potential selfish genetic elements (as is taken for granted, for example, when annotating loci with repeatmasker, we just simply assume that TEs are generally selfish). Almost all other "endogenous" selfish loci were identified from fortuitous genetic observations. Without knowledge of RNAi targeting, these are simply uncharacterized genes, whose knockout mostly likely would have not phenotype. If it is true that they are selfish genes, they activity would only be seen under conditions of derepression/misexpression. Our study lays a foundation for many future studies, but we highlight the value of this novel forward molecular strategy to nominate candidate selfish genes for study.

While many targets of novel hpRNAs bear these signatures, in the revised manuscript, we mostly highlight these points in the discussion section, and only focus on the asymmetric increase in targets of hpRNAs from our comparative analyses in two closely related species.

To address the “age” of hpRNAs, in terms of estimating the birth of hpRNAs discussed in our study, we have done our best to include both near (*D. mauritiana* and *D. sechellia*), and far outgroups (*D. yakuba*) to unambiguously estimate the birth of novel hpRNAs described in our study with the available long-read genome data. Additionally, as suggested by this reviewer (point 3), we also searched two non-reference *D. melanogaster* long-read assemblies to rule out absence of evidence from the *D. melanogaster* reference strain. So, we disagree that we estimated a “rough” age, we know they have to have been born within the simclade Drosophilid ancestor - ie very recently. Clearly, the availability of more within-species long-read assemblies will help us resolve hpRNA evolutionary dynamics better, and address confounding parameters in age estimation, such as gene flow, which is known to occur within the *D. simulans* clade. However, we cannot do any better to pinpoint the evolutionary birth of these hpRNAs, there are no other known species alive that are available for such analysis.

Major points
1) The analysis of the dysregulation of hpRNAs in dcr-2 mutants has some areas that I think could be improved.

i) RNAseq- it is immediately notable from Fig 1 A and B that there is a lot more variance in the changes that occur in simulans compared to melanogaster. This is likely technical rather than indicating increased number of dcr2 dependent transcripts: even miRNA precursors show much greater scatter in simulans. With this in mind, could the authors plot the Z score on the y axis instead- this will take into account the increased overall variance and would enable a clearer comparison of the extent to which hpRNAs are perturbed in the two species.

We thank the reviewer for raising this important point. We do not think that high variance in *D. simulans* is a technical artifact, but likely a consequence of the more severe cytological phenotype in *dcr2* mutants in *D. simulans* compared to D. melanogaster (we also address this point in comment #2 raised by reviewer 3). We previously described complete sterility and failure of spermatogenesis in *dcr2* knockout testes in *D. simulans* (Lin et al. 2018), which likely contributes to a larger set of differentially expressed genes we observe in *D. simulans* compared to *D. melanogaster* (Supplementary Figure 1).

To address variance in the datasets, in our original submission Figures 2A and 2C, we used the LFC shrinkage (“normal” method) in the DEseq2 package for moderated estimation of fold change and dispersion (Love et al. 2014). Briefly, the LFC shrinkage method looks at the largest fold changes that are not due to low counts and use these to inform a prior distribution. So, large fold changes with low dispersion and high statistical information are not shrunk, while imprecise fold changes are shrunk.

In addition, we performed differential expression analyses using three other methods: 1) without LFC shrinkage, 2) a method that implements LFC shrinkage by the “apeglm” method implemented in Zhu et al. (2018), and 3) calculating a Z-score difference, as suggested by this reviewer. In all three cases, we see higher variance and number of genes with significant fold-changes in *D. simulans* (in both directions) compared to *D. melanogaster*. For example, in *D. simulans*, 998 genes show 2-fold upregulation at FDR < 0.01, while in *D. melanogaster* at the same level (2-fold upregulation, FDR < 0.01), only about 290 genes are upregulated. We also wish to highlight that all hpRNAs are among top de-repressed transcripts with high statistical significance and low variance among replicates (normalized counts data per replicate provided in the Supplementary Tabes 1 and 2).

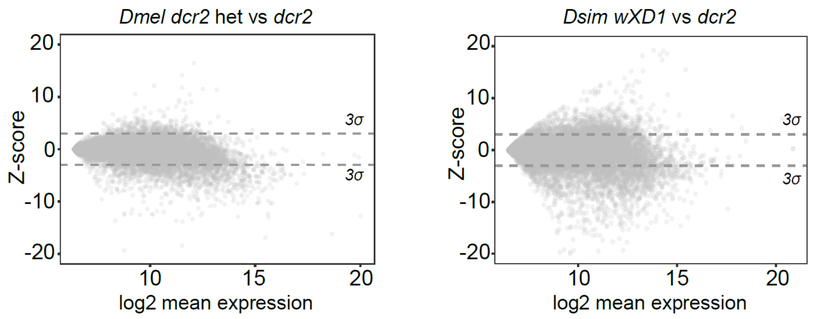

Finally, to address the specific point this reviewer raised, we applied the Z-score method for differential expression analysis between WT and mutant genotypes, but we observed a similar higher variability in *D. simulans*. We provide an example figure (right) for the

referee showing the Z-score difference method, where we used the gene-wise dispersion estimates calculated from DEseq to obtain Z-scores. The higher variability in *D.simulans*, as discussed above, is likely due to the underlying biological effect, and not a technical artifact. However, we found that the Z-score method introduces a technical limitation, partly due to the left-skewed distribution of the RNA-seq data, where deriving meaningful differences in SD from the mean for low-expressed genes is unreliable. Overall, we conclude that the potential reason for the higher magnitude of changes in differentially expressed genes in *D.simulans* is likely due to the nature of developmental defects in *dcr2* mutant in this species.

Having explored all these different ways, because of the potential confusion as to why the lowest values show the least changes in the variance-minimized plots, we decided to go with plotting the data without variance stabilizing transformation in the revised figures. A certain amount of heteroskedasticity is evident when plotting without variance minimization (Love et al 2014), but we thought this is a more intuitive plotting of the data.

ii) Small RNA seq was normalized to spike in, which is great. However, the normalization was not very well described and I struggled to understand exactly what they did. The materials and methods describe normalizing to 52 sequences- is this normalizing separately to each and then averaging, or averaging across the 52 to get a single value for the spike in and then normalizing to this? The two are not equivalent.

We have expanded on the details of how normalization was performed using spike-in sequences in the small RNA libraries. We used the QIAseq miRNA Library QC Spike-In kit for spike-in controls. As indicated in the manufacturer's protocol, our libraries represented ~1-3% of total sequenced reads per library. The normalization was performed across the 52 sequences to get a single value as indicated in the manufacturer's protocol as follows:

$$TPM = \frac{\text{\# of spike\_in reads}}{\text{Total Reads}} \text{ X } 1{,}000{,}000$$

The spike-in reads normalized to a million in the small RNA library was then used to normalize small RNA reads mapped to the loci of interest. TPM normalization of small RNA libraries using the QIAseq miRNA Library QC Spike-In kit is a standard method for spike-in normalization. In addition, according to manufacturer's protocol, after this simple normalization, we did correlation matrices for sample-to-sample comparisons. As indicated in the kit's protocol, we observed a good sample-to-sample correlation for spike-ins (R^2 ranging from 0.95 – 0.99). The kit indicated that if samples deviate from high correlation, then technical outliers (individual spike-in reads) should be excluded from downstream analysis. However, in our case, all samples had high correlation for sample-to-sample comparison (as an example, we show a comparison between *Dsim w[XD1]* replicate 1 vs *Dsim Dcr2* replicate 1). In the example figure, the X and Y axes represent the number of spike-in reads per million reads in Dsim *w[XD1]* and Dsim Dcr2 genotypes. The black dots in the graph represent individual spike-in reads (52 total). So, for all downstream analyses (e.g., bigwig for visualizations), we used the TPM normalization method.

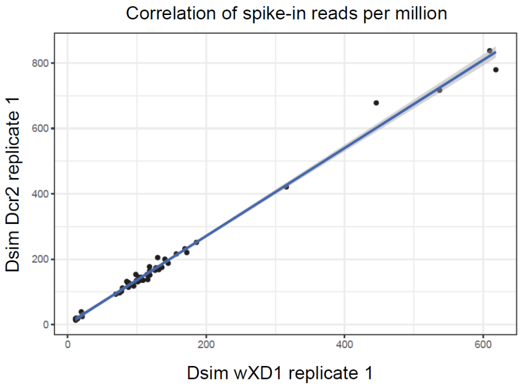

Moreover, this normalization method will transform the data into a continuous distribution BEFORE normalization, which will lead to poor performance for low expression transcripts. An alternative would be to create a full counts table just for the spike transcripts and generate size factors via DESeq from this, and use this for normalization.

As suggested by the reviewer, we also estimated size factors via DEseq from spike-in reads. The scaling factor from the estimated size factors is approximately equal to the TPM normalization described above from the QIAGEN kit. For example, for the dcr2_het_rep1, the estimated size factor from 52 spike-in reads in the library is 1.94. So, for a locus with ~10000 reads, multiplying by the scaling factor would give a normalized value of 19400. The TPM method gives 22225 reads. So, we decided to use the standard TPM normalization method which is routinely used in small RNA normalization with spike-in sequences.

More information about the sRNAs would also be useful- could the authors please expand Figure 1 to contain MA plots for sRNAs and boxplots comparing fold change in miRNAs to fold change for hpRNAs.

We thank the reviewer for this suggestion, however we feel the bar graphs show the magnitude of change much better (when plotted with normal values, as opposed to log2 fold on an MA plot). While the MA plot is helpful to get a general overview of the entire dataset, when we focus in on the relatively small set of hpRNAs and miRNAs, the box plot and bargraphs allow the reader more direct visualization of each value and its comparison between mutants.

2) I think that there is something missing from the logic that targets of youngest hpRNAs are the most dysregulated. If I am not mistaken this is based on finding the targets of the hpRNA derived siRNAs; however it is possible that some of these genes might be targeted by other siRNAs as well, which might also be dysregulated in the dcr-2 mutants. This could confound the conclusion because it might be the changes in the other small RNAs that is responsible for the changes in expression rather than the changes in hpRNA (i.e. these genes might not even be functional targets of hpRNA). I think this is unlikely to be a confounder but it would be good if the authors could rule it out. My suggestion would be to specifically select a subset of the targets of hpRNA that have sequence similarity to hpRNA but NO other small RNA (including miRNA seed matches). Then reproduce their age analysis on this subset. I think it may be enough data for them to reproduce their observation; if this now shows no clear difference then it might indicate that their hypothesis that hpRNA changes are responsible could be wrong or incomplete.

We wish to address this comment "*however it is possible that some of these genes might be targeted by other siRNAs as well, which might also be dysregulated in the dcr-2 mutants."* through the lens of our prior siRNA work and also the history of antisense gene suppression. While this is theoretically possible, and thus may not be ruled out categorically, in a practical sense it is doubtful that strong effects would be seen due to co-targeting by other siRNA classes. This sense comes from our work and from the general RNAi community (including the leading siRNA biochemistry labs), which strongly support that intermolecular dsRNA (eg cis-NATs) is far less potent than intramolecular dsRNA (eg hpRNA) in conferring target repression.

In flies, our prior efforts on siRNA networks uncovered both intramolecular and intermolecular substrates for the RNAi: 1) inverted-repeat containing hpRNAs that can be a substrate for siRNAs (Okamura 2008 Nature), and 2) cis-NAT (cis-natural antisense transcripts) loci, which can generate double stranded RNAs that can be processed into siRNAs by *dcr2* (Okamura 2008 NSMB). We previously reported that cis-NAT-siRNAs seem to confer mild regulation at best; in fact, we could not definitively show evidence of their regulatory activity (Okamura 2008 NSMB). This probably relates to the fact that formation of in vivo dsRNA is a limiting factor in generating an effective trigger, and why "RNAi" is a preferred method while antisense RNA injection was never reliable. In any case, there is not overlap in hpRNA-targets with genes that produce cis-NAT-siRNAs (which might putatively be auto-targeted).

The other relevant point ("*targets of hpRNA that have sequence similarity to hpRNA but NO other small RNA (including miRNA seed matches)*") is that siRNAs associate with the effector AGO2, which is incapable of regulated target genes via seed matches. This only applies to miRNA guides in AGO1, for which the capacity for non-slicing regulation is mediated by obligate association with GW182/TNRC6, which subsequently recruits deadenylation machinery and/or interferes with cap-dependent translation. In fact, to our knowledge it is unknown what the full range of AGO2-mediated target complementarity is, but it cannot regulate via seed matches.

We hope that this is an appropriate discussion for the referee.

3) The conclusion that hpRNAs that are not shared in simulans and melanogaster are youngest is broadly true but in specific instances may be incorrect due to polymorphism in melanogaster meaning that some hpRNAs apparently missing in melanogaster are just not present in lab drosophila. It would be relatively straightforward for the authors to refine this set by searching the entire melanogaster collection for this subset of hpRNAs using blast and discarding the few that are in fact found in some dmel strains- I think this would be quite a good step to take.

To rule out incorrect age estimation due to segregating polymorphisms for novel hpRNA loci (from D. simulans) in *D. melanogaster*, we performed a homology search for all novel hpRNAs in two *Dmel* long-read genome assemblies from Chakraborty et al. 2019 (A6 and KSA2 strains). Homology searches in additional long-read *Dmel* genome assemblies corroborate our earlier findings that the novel hpRNAs indentified in *Dsim* originated in simulans clade. They are truly inserted within the syntenic regions of ancestral fly genomic sequences . These observations generalize what we have already reported recently for birth of Dox family hpRNAs in the simulans clade (Vedanayagam, NEE 2021).

Minor
All names of species should be written out in full. It's quite difficult to follow otherwise, especially when many other species are introduced (D yakuba etc)

We appreciate the reviewer concern. When we first introduce the species name, we include the full species name with abbreviation. For example, *D.simulans* (*Dsim*) etc. We have used these abbreviations previously (Chakraborty et al. 2020, Vedanayagam et al. 2021), particularly for the ease of representing species names in figures. Also, as the centerpiece of this manuscript is a species-level comparison, we callout *D. melanogaster* and *D. simulans* plenty of times, and we feel the abbreviation is appropriate for reducing character space in the manuscript. However, we are amenable to further editorial input on this.

MA plot needs to be explained in the main text {p6}

We included a following sentence in the manuscript to briefly explain the MA plot: " We plotted differential expression of *Dmel* and *Dsim* RNA-seq data in MA plots (**Figure 2A-D**), which display the log-ratio of *dcr2 vs*. wild type controls of RNA-expression (M-axis) and the average gene expression in the RNA-seq data (A-axis)."

Fig 1 E and F axis do not stretch far enough up.

We modified the figure panels.

Why is there no MA plot for the siRNAs? I think this would be worth including (see above).

As outlined in the response to comment #4 in the major points, we preferred bar graphs to MA plots, as the bar graphs currently included in figure panels 2 E-G show the magnitude of change between the wildtype and the mutant genotype much better, especially when plotted with normal values, as opposed to log2 fold on an MA plot.

"These were highly confident" p7 should be "these annotations were high confidence"

We changed this in the main text.

"and not simply by homology"- p8: didn't make sense to me but I think the authors mean "and not simply by sequence complementarity to hpRNA derived siRNAs" unless I misunderstood.

We edited the text to state "and not solely on the basis of hpRNA/target homology", to reflect we are not only going on sequence complementarity but by target derepression in dcr2 mutants.

“The "oldest" Dsim hpRNAs (i.e., still relatively young, but shared with Dmel)” is a very strange phrase; I suggest clarifying “D. simulans hpRNAs that were shared with D melanogaster

We clarified this statement in page 13 to state “*Dsim hpRNAs that are conserved in Dmel..*”

“broadens the scenario that the Dsim X” p13 not clear what this means

We repharased the sentence to clarify that there is a disproportionate contribution of the X chromosome to potential ongoing genomic conflicts suppressed by hpRNA/RNAi pathway in *Dsim*. We also added a note highlighting whether the novel hpRNA targets in *Dsim* may be in conflict between X and the Y, like the *Dox* family, will be investigated in future studies.

“described in detail about the existence” p 13 should be “described the existence…”

We changed this in the revised manuscript.

Reviewer #2: This manuscript presents evidence for an expanding network of novel hairpin RNAs in Drosophila simulans and speculates that these data reveal the importance of endogenous RNAi for intrachromosomal conflicts and speciation. The authors utilized mutants of Dicer-2 in both D. melanogaster and D. simulans to perform differential expression analysis of testis total RNA and testis short RNA sequencing libraries; in dcr-2 mutants, loss of siRNAs along with accumulation of their pri-hpRNAs and upregulation of genes containing sequences homologous to those siRNAs allowed them to identify hpRNA-target gene interactions. This analysis yielded only one novel hpRNA in D. melanogaster but revealed 21 novel hpRNA in D. simulans, and all 21 seem to be de novo acquired in the sim-clade (they are not found in closely related outgroups). Further, most of the D. simulans hpRNA target genes reside on the X chromosome, suggesting these pairs could be involved in a sex ratio distortion conflict.

Major Points:

1) The data and analyses support the argument that D. simulans has a "young" set of hpRNA-target interactions--with a bias for X-linked targets--that exhibit stronger repression relative to conserved "older" hpRNA-target pairs. However, no experimental evidence is provided to buttress the claim that any of the novel hpRNA-target pairs are de facto examples of a meiotic drive conflict. Based on the data presented, the authors could soften some of the language drawing conclusions about the connection between these newly identified hpRNAs and sex chromosome drive. Alternatively, the authors might produce additional data to support this argument. For example, a knockout of hp-88 (or hp-ballchen) could be made to test if a drive is unleashed.

In the revised manuscript, we moved the description of novel hpRNAs and their potential connections to sex chromosome conflict to the discussion section. We agree with the reviewer that besides *Dox* family (for which we have generated knockouts of *hp-Nmy* and *hp-Tmy*), whether or not novel targets of hpRNAs are putative meiotic drivers remain to be tested. Efforts to generate knockouts at other novel hpRNA loci in the future may reveal drive properties of novel targets, and we have extensively modified the language in the revised manuscript to focus only on the asymmetric increase in novel hpRNA loci from our comparative analyses. It is a substantial effort to generate knockouts in this non-model species, and we believe this is definitely interesting but appropriate for a future study.

2) The “connections to transposons” seems tenuous. Besides one hpRNA that may target BS2/Jockey, is there additional evidence for the connection to TE biology? Is BS2/Jockey upregulated in dcr-2 mutants? BS2/Jockey does not appear to be in Figure 6.

We have now modified the statements regarding connections to transposable elements (TEs) in the revised manuscript. In the animal germline, transposon regulation is under the control of a distinct small RNA pathway called piwi-interacting RNA (piRNA) pathway, and piRNA pathway mutants exhibit severe

developmental defects, in conjunction with global TE misregulation. Although TE mapping siRNA size class (~21-22nt) small RNAs are detected in our libraries, *dcr2* mutants do not exhibit global TE misregulation signatures like the piRNA pathway. In our case, *hp-ODox-2* contains an inverted repeat homologous to BS2/Jockey. However, as small RNA libraries also have abundant piRNAs mapping to BS2/Jockey elements, BS2/Jockey elements do not show derepression in *dcr2* mutant. In dcr2 mutants, we have observed upregulation of gypsy family TEs, but potential roles of siRNAs in regulating gypsy family remain unclear. We haven't included BS2/Jockey as a target in figure 6, as unlike regular genes/protein-coding targets, BS2/Jockey elements occur in thousands of copies in the genome. Due to their widespread genomic presence, including centromeres (Chang et al. 2019) we excluded BS2/Jockey in Figure 6. We have added this information in the figure legend.

3) More generally, the structure of the writing can be improved. Multiple sections are difficult to follow for an audience not already very familiar with the specifics of this system. A few examples are highlighted below.

"Unexpected complexity in the Dsim hpRNA network related to Dox family loci"
First, referring to the inferred ancestral ODox as well as the contemporary putative first derivative of ODox both as ODox is confusing. Second, Figure 3B appears to illustrate hp-ODox1 and hp-ODox2 identically. A sequence alignment (or cartoon alignment) would be more helpful for the reader to understand their similarities and differences.

"Innovation of Dsim hpRNAs that target other HMG-box loci"
Is the argument being made here that HMG box factors are often targets of hpRNAs? If so, it would be helpful to state this clearly up front and briefly provide evidence for the claim; leave the explanations of the complexities of these hpRNAs for a later paragraph. Additionally, there is a cumbersome explanation of how tHMG is not in the MST-HMG subclass but is related protamine, which is possibly evolving rapidly, and the overall point of this opening paragraph is unclear. Although the complexity of the tHMG-related hairpins and targets it appreciated, it seems odd that the authors do not mention that both tHMG2-Xhet-1 and tHMG2-Xhet-2 are among the most highly up-regulated Dsim-specific targets (Fig. 6C). These data appear to highlight an intra-chromosomal conflict.

We thank the reviewer for the suggestion to improve writing for clarity. We rearranged the introduction for this section of the manuscript so as to reach readers who are not already familiar with the specifics of this system, including the sections outlined above by the reviewer. To clarify the relationships between the tHMG loci and Protamine derived Dox family genes, we have included a new phylogeny showing their relatedness in new **Supplementary Figure 6**.

"Functional repression by hpRNAs is most overt for the youngest siRNA loci"
Some of these results may be overstated. For example, the increased expression of tapas is below the 1.5-fold threshold (Fig. 6D) yet is cited as supporting evidence for functional repression by hp-ODox1. Additionally, the increased expression of krimp is almost as high as increased expression of tapas despite the lack of siRNA targeting krimp, as noted previously (Fig. 3D and earlier in the text). Finally, is the comparison to miRNA appropriate?

We thank the reviewer for pointing out this discrepancy. The tudor factor Tapas is only target of novel hpRNAs in Dsim for which we do not detect upregulation > 1.5 fold despite detecting antisense piRNAs that emerge from hp-ODox-1. We highlighted this exception and added a note in the *"Functional repression by hpRNAs is most overt for the youngest siRNA loci*" section.

We feel comparison to miRNAs and the magnitude of changes in miRNA targets appropriate as at least two decades of functional studies have provided evidence that miRNA targets barely change upon knockdown for many miRNA loci. One of the highlights and importance of study is how this convention is flipped for hpRNA/RNAi regulation, as conserved targets of hpRNAs barely change, but the targets of novel hpRNAs exhibit most overt changes.

Minor Points:

Page 4, last paragraph of Introduction: Is it surprising that young hpRNAs would have would have a strong effect in a conflict scenario?

We believe it is indeed surprising, because it has never been described to this scale, beyond 2 examples (Nmy and Tmy). And we emphasize that it might not have been the case at all: there might not be any other new hpRNAs, or new hpRNAs might not have had any targets (as is proposed for new miRNAs), or young hpRNAs might not have detectable regulatory effects on these targets, etc. In retrospect, it makes sense, (*that young hpRNAs would have would have a strong effect in a conflict scenario*) and we hope that publication of this study can bring this to light as a general principle and a canonized conclusion.

Page 9, second paragraph: Typo "functiosn" should be functions.

We corrected this typo.

Page 11, second paragraph: CG17358 does not agree with Figure 5B (CG17385). There are other instances of gene numbers in the text, tables, and figures not matching. Please check them for consistency.

Thank you for careful reading. CG17385 is the correct name, and we corrected this typo in the manuscript. We also did a thorough check for matching for gene names between the manuscript and tables/figures.

Page 14, first paragraph: There may be a typo in "the miRNA pathway does not typically does adaptive targeting".

We modified the sentence to read as "*the miRNA pathway is not typically involved in adaptive targeting*"

Page 14: The explanation of protamines as they relate to genomic conflict may be more useful in the introduction. Additionally, it is not clear that data from this manuscript provides any new information in regards to piRNAs or TEs, so these paragraphs may not be relevant here.

We included a description of protamines and their potential roles in genomic conflict in the introduction. With regards to piRNA pathway, our study uncovered fragments of piRNA pathway factors embedded in hpRNA-loci that can target these factors via siRNAs. While intriguing, future functional tests are needed to uncover the regulatory effects of hpRNA siRNAs in silencing piRNA factors, and the broad co-operative or antagonistic relationships between the two small RNA pathways in regulating germline conflicts. As suggested by the reviewer we removed the reference to piRNA factors and TEs in the discussion sub-heading. However, we wish to highlight the emerging theme from other studies where piRNA pathway factors may be involved in meiotic drive (for e.g. *HP1D2*, a paralog of a key piRNA pathway gene called *Rhino* in D. simulans). In our case, while homology of hpRNA-siRNAs to piRNA factors Tapas and Krimper may reflect a potential conflict scenario, however, it remains to be determined *how* and *why* these piRNA factors are integrated into the siRNA regulatory network. We discuss this possibility in the revised manuscript and highlight potential crosstalk between the two small RNA pathways emerging from recent studies.

Page 15, last paragraph: "…active genomic conflict scenarios that must be playing out in these species" should probably be worded as "may" be playing out in these species.

We corrected this sentence in the revised version of the manuscript.

Page 16, last paragraph: This seems to be a typo "{Lin, 2018 #14350."

This is a reference insertion error, we fixed this.

Figure 3 caption: Another example of conflicting gene numbers: GD1739 or GD17329?

Thank you again for close reading. We corrected the conflicting gene name in the Figure 3 caption.

Figure 3 caption: (C) It is unclear what is being referenced with the text "and other regions of hp-ODox1 bear TE sequences."

We meant to indicate that in addition to homology to Tapas, the inverted repeat arms of *hp-ODox1* also bear homology to TE sequences. We corrected this in the figure 3 caption C.

Figure 6 caption: "(D)" is missing.

We added caption D to Figure 6.

Reviewer #3: This is a very nice overview of the dynamics of hpRNA evolution in the simulans clade. Using new Dicer alleles in simulans, the authors uncover a complex web of loci that interact through endogenous RNAi, presumably part of sex ratio conflict. I think the manuscript could be somewhat improved by providing, for figures 3,4 and 5 a) more context and b) alignments that support the models in figures 3,4 and 5. In particular, for figure 3, rather than simply presenting the model for the generation of Odox followed by other Dox variants and hp-ODox1,2, the model would be supported by combining phylogenetics. Otherwise, it is not clear if the model they propose is suppported. 3A and 3B are a models for the evolution of the Dox network, but it is not supported by data that is presented to the reader.

We thank the reviewer for the positive comments on the manuscript. We expanded the text in the revised manuscript to provide more contexts from our previous study on Dox superfamily evolution (Vedanayagam et al. 2021). Figure 3 pertains to the contemporary state of ancestral Dox family expansions that further spawned the meiotic drivers MDox and Dox. In Vedanayagam et al. 2021 we discuss the evolution and phylogenetic relationships of Dox superfamily sequences and we provided additional details in the revised manuscript referring to our previous study. We provided additional phylogenetic relationships of the all testis HMG-box containing genes, including the tHMG loci discussed, and the relationship of ancestral ODox (and contemporary hp-ODox-1 and hp-ODox2) to Dox family genes in the new **Supplementary Figure 6**.

My only real concern is that many of the statements are not supported by statistical analysis. For example, they make the claim that new sim hpRNA targets are enriched on the X, but not new hpRNA loci. By my count, for figure 7, this is what they have for sim specific loci:

new hpRNAs: X: 6, Autosomes: 11
new targets: X: 11, Autosomes: 4

By a Fisher's exact, the p-value is 0.0416. So, this is significant enrichment. They should mention this.

Thank you for performing this test. Yes, we should definitely mention this!

Please perform additional statistical analyses when evaluating:

1. The enrichment of differentially expressed hpRNA loci in sim vs. mel based on the results of 2A and 2C.

We performed two-tailed Fisher's exact test for enrichment of differentially expressed novel hpRNAs in Dsim vs. Dmel shown in Figures 2A and 2C and the p-value is 0.0010. We have included this test in the manuscript and figure2 legend.

2. The difference for fold expression change in 6C vs. 6B. The claim is that the new sim hpRNA targets are more sensitive to dicer loss, but this isn't statistically evaluated.

We performed a Weltch t-test (two-tailed) to compare fold expression change between conserved vs. novel Dsim hpRNA targets, and we find that the targets of novel Dsim hpRNAs are significantly de-repressed compared to conserved Dsim hpRNA targets p-value = 0.016. We included this test in the revised manuscript.

I have two other questions/requests.
1. Please provide an analysis of target/hpRNA %identity between 6C and 6B. Is there simply more sequence identity between sim specific hpRNAs and targets? Related, please provide quantitative data for the abundance of small RNAs that are antisense to each of the respective targets in 6C and 6B. There may be a quantitative difference that would be important to present to the reader.

We have included the overlap (in bp) between targets (figure 6) and their cognate hpRNAs (figure 2) in the Supplementary Table 1, under the column "target overlap". We added a new supplementary table (Supplementary table 5) to provide abundance of antisense small RNAs to hpRNAs targets shown in Figures 6B and 6C).

2. Please provide a photo of the dicer mutant sim testes compared to wildtype and Dicer hets. It will be important for the reader to see these images to evaluate the results in 2C. If the testes are significantly atrophied, this might explain the greater variance in baseline log2 fold change between sim and mel.

We have thoroughly documented testis morphology in RNAi mutants (dcr2 and ago2) in *D. melanogaster* (Wen et al. 2015) and in *D. simulans* (Lin and Hu et al. 2018), and these studies are cited. These papers report that Dmel RNAi mutants can complete spermatogenesis and are fertile (although abnormal), whereas Dsim RNAi mutants have much more severe defects in spermatogenesis and do not deposit any mature sperm in the seminal vesicle. These data come from hand-dissected testis, which is the relevant tissue. Based on these papers, it is fully expected there will be greater overall gene expression changes in Dsim testis for this reason. We discuss this to address a similar point raised by reviewer 1 (major point #1).

Data availability:
RNA-seq, small RNA, 5'seq, and 3'seq data from Dmel and Dsim described in this study are available in the GEO database: GSE230111.

Have all data underlying the figures and results presented in the manuscript been provided?
Large-scale datasets should be made available via a public repository as described in the PLOS Genetics data availability policy, and numerical data that underlies graphs or summary statistics should be provided in spreadsheet form as supporting information.
Reviewer #1: ***No:*** *The authors haven't yet released these data.*
Reviewer #2: Yes
Reviewer #3: Yes

PLOS authors have the option to publish the peer review history of their article (what does this mean?). If published, this will include your full peer review and any attached files.

If you choose "no", your identity will remain anonymous but your review may still be made public.

Do you want your identity to be public for this peer review? *For information about this choice, including consent withdrawal, please see our Privacy Policy.*
Reviewer #1: No
Reviewer #2: No
Reviewer #3: No
